# Prevalence and Clinical Management of Adrenal Tumour-Related Hyperandrogenism: A Narrative Review

**DOI:** 10.3390/life14030360

**Published:** 2024-03-09

**Authors:** Sanja Medenica, Dusan Zivanovic, Domenico Milardi, Carmine Bruno, Ljubica Batkoska, Emanuela Traini, Alfredo Pontecorvi

**Affiliations:** 1 Department of Endocrinology, Internal Medicine Clinic, Clinical Center of Montenegro, School of Medicine, University of Montenegro, 81000 Podgorica, Montenegro; 2 Clinic of Endocrinology, Diabetes and Metabolic Disorders, University Clinical Center of Serbia, 11000 Belgrade, Serbia; 3 Operative Unit of Internal Medicine, Endocrinology & Diabetes, Fondazione Policlinico Universitario A. Gemelli IRCCS, 00168 Roma, Italy; 4 Dipartimento di Medicina e Chirurgia Traslazionale, Università Cattolica del Sacro Cuore, 00168 Rome, Italy; 5 Istituto Dermopatico dell’Immacolata (IDI IRCCS), 00167 Rome, Italy; 6 Faculty of Medicine, SS. Cyril and Methodius University of Skopje, 1000 Skopje, North Macedonia; 7 Endocrine Surgery Unit, San Carlo di Nancy—GVM Care & Research Hospital, 00167 Rome, Italy

**Keywords:** adrenal, testosterone, hirsutism, acne

## Abstract

Hyperandrogenism is a condition in which the levels of androgen hormones in the blood are significantly increased and could be of an adrenal or ovarian origin. The adrenal androgens, normally secreted by the zona reticularis, are steroid hormones with weak androgen activity. The causes of hyperandrogenism are diverse and could be endogenous and exogenous. Androgen excess affecting different tissues and organs results in clinical features such as acne, hirsutism, virilization, and reproductive dysfunction such as oligomenorrhoea/amenorrhoea. Although androgen excess is rarely associated with adrenal tumours, it is important as it could be predictive of malignancy. A careful evaluation of the androgen pattern, also in patients with clear signs of hyperandrogenism, could be useful. Laboratory evaluation should focus on measuring total testosterone levels, followed by the estimation of other androgens such as dehydroepiandrosterone and androstenedione, and using visualisation procedures in the further management. The treatment of adrenal hyperandrogenism is eminently surgical, in consideration of the frequent malignant origin. The aim of this review is to elaborate and summarize the prevalence and clinical management of hyperandrogenism of an adrenal origin by describing the physiological mechanisms of adrenal androgen steroidogenesis, the clinical manifestations of hyperandrogenism with a special reference to hyperandrogenism in adrenal adenomas and carcinomas, and the diagnostic methods that will lead us to establishing the correct diagnosis and different treatment options to manage this condition according to the clinical presentation of the patient.

## 1. Introduction

Hyperandrogenism is a condition characterized by increased levels of circulating androgens or augmented sensitivity of target tissues to them [1]. Androgens are steroid hormones that are primarily produced by the testis, ovaries, and adrenal glands, in males, females, and in both sexes, respectively; therefore, androgen excess (AE) arises from abnormal ovarian or adrenal sources [1,2]. Adrenal androgens, normally secreted by the zona reticularis, present weak androgen activity; their action in fully androgenized adult men seems to be negligible, while representing the major androgenic source in adult women and in both sexes before puberty [1]. The most abundant adrenal androgens are dehydroepiandrosterone (DHEA) and its “reservoir” molecules DHEA sulphate (DHEA-S) and androstenedione (Δ4-A). The production of testosterone (T) by adrenals is minimal. DHEA and DHEA-S are mainly the products of zona reticularis, and Δ4-A and testosterone are secreted by both zona reticularis and zona fasciculata [3]. Adrenal androgens are secreted in small amounts during infancy and early childhood, and their secretion gradually increases during puberty, paralleling the growth of the zona reticularis, which continues through the third decade of life until a gradual decline starting at the fourth decade: this phenomenon is commonly named as “adrenopause” [1,3,4], although this term is improper and obsolete, since a decline in DHEA production is linear with no break, with no “menopause-like” switch.

A patient with hyperandrogenism presents with a characteristic clinical picture, including irregular menstrual cycles, clitoral hypertrophy, virilization, and change in the secondary sex characteristics (voice pitch, hair pattern), polycystic ovaries, cystic mastitis, infertility, and metabolic syndrome [5]. A clinical example could be the so-called “hyperandrogenism, insulin resistance (IR), and acanthosis nigricans (HAIR-AN) syndrome” that is considered a subtype of polycystic ovary syndrome (PCOS) [5,6]. With reference to hair follicles, androgens have a variety of physiological and pathological impacts on the skin [2]. The term hyperandrogenism refers to all possible androgen-associated skin conditions that occur due to the increased local androgen effect on the skin. The most prevalent clinical signs of hyperandrogenism are hirsutism and acne, either as a single manifestation or in combination [5,7,8]. Hirsutism affects 5–15% of the women and is the most commonly used clinical diagnostic criterion of androgen excess or hyperandrogenism [2], although its presence should be carefully evaluated also in relation to ethnicity of patients, in order to avoid false positives.

The causes of hyperandrogenism are diverse and are divided into two groups: endogenous and exogenous. The endogenous factors are also divided into two groups: endogenous factors with neoplasia and endogenous factors without neoplasia. Endogenous factors with neoplasia include androgen-producing benign and malignant neoplasms of the ovaries and adrenal glands (adenomas and carcinomas), the pituitary gland, and the hypothalamus, as well as paraneoplastic syndromes, such as the production of the adrenocorticotropic hormone (ACTH) by a bronchial carcinoma. Endogenous factors without neoplasia can occur due to congenital anomalies, such as the congenital adrenal hyperplasia (CAH) [4] and XY disorders and lifestyle disorders, for example, stress, anorexia nervosa, and endocrine disorders (PCOS, Cushing disease, hyperthyroidism, etc.). Exogenous factors can also be involved, especially hormonal contraceptives such as progestogens with an androgenic effect, androgens or anabolic steroids, antiepileptics, corticosteroids or ACTH, metyrapone, and certain cosmetics [4].

Many diseases of nonreproductive organs display sex bias, and the adrenal gland is no exception as a lot of adrenal cortex diseases, including adrenocortical tumours, are more prevalent in women than in men [9]. Puberty has a strong effect on the amplitude of sexual dimorphism, as it has been shown that DHEA elevation is not only caused by ACTH, but also by luteinizing hormone (LH) stimulation [10]. Furthermore, Marina et al. investigated the relationship between LH and insulin resistance (IR); adrenal tumour size (ATS) and IR; and LH and cortisol and concluded that there is clinical evidence showing that the enhanced adrenal cortex steroid production in female patients with adrenal tumours and mild autonomous cortisol secretion is a result of the synergistic action of LH and insulin, which means that endocrine interactions with gonadal hormones need to be taken into consideration [11].

The aim of this narrative review is to elaborate and summarize the prevalence and clinical management of hyperandrogenism of an adrenal origin by describing the physiological mechanisms of adrenal androgen steroidogenesis, the clinical manifestations of hyperandrogenism with a special reference to hyperandrogenism in adrenal adenomas and carcinomas, and the diagnostic methods that will lead us to establishing the correct diagnosis and different treatment options to manage this condition according to the clinical presentation of the patient. A literature search was carried out through the “PubMed” database, in December 2023, matching the MeSH terms “adrenal”, “hyperandrogenism”, and “adrenocortical tumour”, and selecting current guidelines on the argument and previous original papers and systematic reviews on the topic of the past 10 years. 

## 2. Adrenal Androgen Steroidogenesis

The adrenal gland is a bilateral organ made up of the cortex and medulla [8]. In this review, we are concentrating on the cortex, especially those parts of the cortex that produce androgens. There are three layers that form the adrenal cortex, and they are called the zona glomerulosa (ZG), the zona fasciculata (ZF), and the zona reticularis (ZR). C19 androgens are primarily produced by human AG in the reticular zone and, to a lesser extent, in the fascicular zone [9]. All three layers have cells that are mitochondria-rich and could be distinguished by their cristae shape. Zona reticularis is the inner layer of AG and could be histologically distinguished from the other layers by prominent lysosomes in the cytoplasm. Zona fasciculata has cells with many lipid droplets and abundant smooth endoplasmic reticulum [8]. Adrenal androgen production already starts during foetal development, with the formation of the so-called foetal adrenal zone in the cortex, and in that period of life, the adrenals are almost completely dedicated to androgen production (especially dehydroepiandrosterone (DHEA)) [10]. In adult life, the adrenals produce different C19 androgens such as DHEA, DHEA sulphate (DHEAS), androstenedione (A4), androstenediol, and 11β-hydroxyandrostenedione (11OHA). Their androgen activity in adults is low, but they serve as a reservoir for the production of more potent forms of androgens such as testosterone and oestrogens such as oestradiol [11].

Androgen production in the adrenal glands is controlled by the hypothalamic hormone corticotropin-releasing hormone (CRH) and the anterior pituitary secretion of the adrenocorticotropic hormone (ACTH). ACTH stimulates the adrenal glands’ ability to produce androgens such as DHEA and androstenedione [12]. ACTH binds to its receptor, called the melanocortine 2 receptor (MC2R), which activates cyclic adenosine monophosphate (cAMP) production and calcium ion influx, contributing to protein kinase A (PKA) activation. PKA and Ca2+ cytoplasmatic influx activate transcription factors targeted to modulate the synthesis of the enzymes [9]. Steroidogenesis begins with a cholesterol molecule, which can be formed in adrenal cells from scratch or taken from the circulation. Then, steroidogenic acute regulatory protein (StAR) mediates the translocation of the free cholesterol from the cytoplasm to the inner mitochondrial membrane, where steroidogenesis starts [9]. Within the inner mitochondrial membrane, the sidechain cleavage enzyme (CYP11A1, P450scc) catalyses the conversion of cholesterol to pregnenolone. Pregnenolone is then hydroxylated by the enzyme 17α-hydroxylase encoded by *CYP17A1* to form 17-hydroxypregnenolone. In the reticular zone, in the endoplasmic reticulum, an enzyme encoded also by *CYP17A1* with its 17,20-lyase activity converts 17-hydroxypregnenolone to dehydroepiandrosterone (DHEA), which is converted to androstenedione by 3βhydroxysteroid dehydrogenase type 2 [13]. CYP17A1 is needed for DHEA synthesis from pregnenolone and for androstenedione (AD) synthesis from progesterone. CYP17A1 is present in both ZF and ZR, and its 17,20-lyase reaction is 10 times more activated by the cofactor cytochrome b5 (*CYB5A*), which is not present in the ZF. Sulfotransferase SULT2A1 conjugates DHEA to DHEAS. The adrenal generates little amounts of testosterone, with the action of 17b-hydroxysteroid dehydrogenase type 5 (17bHSD5, AKR1C3) on AD [14]. Dehydroepiandrosterone (DHEA) and its sulfoconjugate are the main androgens secreted from the ZF and ZR [15].

Among CRH and ACTH, there are also other modulators of androgen activity, like insulin and transforming growth factor b (TGF-b). Intra-adrenal production controllers are enzymes and proteins involved in the steroidogenic pathway, specifically 17,20-lyase activity and DHEA sulfotransferase. DHEAS has a longer half-life and thus is more appropriate for assessing adrenal function [16]. As mentioned before, DHEA and DHEAS production are increased during the foetal period, but their levels decrease in early infancy. With puberty, their production increases dramatically again, reaching the highest levels in the third decade of life. With further ageing, DHEA and DHEAS levels decline to 10 to 20% of those in young adults [17].

## 3. Clinical Manifestations of Hyperandrogenism

AE affects different tissues and organs, resulting in clinical features such as acne, hirsutism, virilization, and reproductive dysfunction such as oligomenorrhoea/amenorrhoea [18]. Clinical manifestations of adrenal hyperandrogenism are age- and sex-dependent. Specific clinical manifestations are related to the progression of symptoms and size of the tumour. Androgen-secreting adrenal tumours are rare and usually aggressive and may be associated with high cortisol excess [19]. Adrenal gland dysfunction may contribute to the clinical manifestation of hyperandrogenism in PCOS. 

Independent of the primary source, AE in prepubertal children affects growth and somatic development, and skeletal maturation with premature epiphyseal fusion, leading to short adult height. In prepubertal boys, virilization is presented by penile enlargement, growth of hair in androgen-dependent areas, deepening of the voice, and development of other secondary sexual characteristics, isosexual precocious puberty. In prepubertal females, hirsutism, acne, and clitoromegaly and precocious puberty are the main characteristics [20]. In the pubertal period of life, AE causes the acceleration of puberty and skeletal development in males, as previously mentioned with a repercussion on height, similar as in cortisol excess where the inhibition of the hypothalamic–pituitary–gonadal axis also occurs. It is similar in females, accompanied with virilization and amenorrhoea [20]. In the adult period, more clinical manifestations are evident in women, causing hirsutism, acne, male-pattern baldness, menstrual irregularities, oligomenorrhea or amenorrhea, infertility, and even frank virilization. Differently, for adult males, acne and hirsutism are considered rare, while testis volume, testosterone levels, and spermatogenesis may be impaired [18]. As terminological aspects are concerned, a difference between hirsutism and virilization should be made: the first one is defined as androgen-dependent excessive pattern hair growth; in virilization, androgen levels are sufficiently high to cause additional signs and symptoms, such as deepening of the voice, breast atrophy, increased muscle bulk, clitoromegaly, and increased libido; in particular, clitoromegaly is diagnosed when the clitoral index, calculated as width (mm) × length (mm), is >35 mm^2^. The presence of virilization, suggesting the possibility of ovarian or adrenal androgen-secreting neoplasia, although a congenital onset, is typical of classic CAH, which rarely can be presented in adulthood [20]. Acne is frequently associated with hirsutism but is not always a sign of hyperandrogenism. Androgens directly affect the pilosebaceous unit and have an important role in acne development. The prevalence of AE in women with adult acne varies between the studies, and in the largest study, 55% of 835 women with adult acne had increased androgen levels [21] and it may be estimated that the prevalence of hyperandrogenism acne is about 50% in adults [22]. Female adult acne may be associated with hirsutism and a prevalence of hirsutism in women with adult acne is about 20% to 30% [23]. Although it is a marker of insulin resistance and is rarely seen with androgen-secreting tumours, acanthosis nigricans is a mucocutaneous condition characterized by velvety hyperpigmentation patches that appear mainly on the skin of the base of the neck, axilla, antecubital fossae, and groin [24]. AE could be associated with adverse chronic health issues such as impaired glucose intolerance, nonalcoholic fatty liver disease, and cardiovascular disease, especially in PCOS, Cushing disease, syndrome of severe insulin resistance [25,26,27]. 

**Figure 1:**
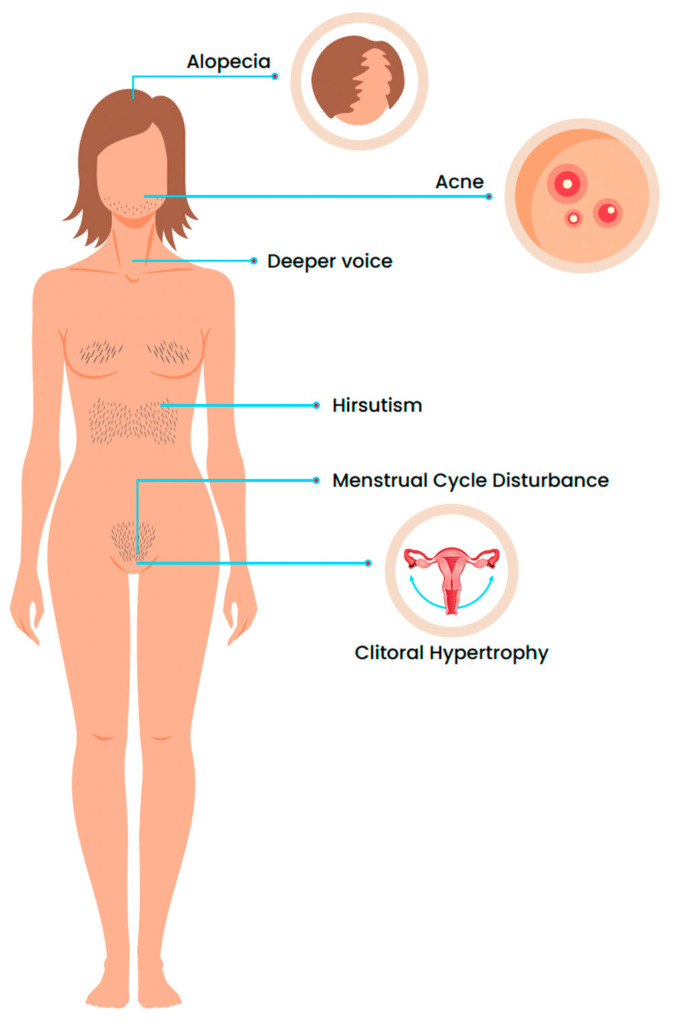
Clinical features of adrenal hyperandrogenism.

Hyperandrogenism after menopause is a rare condition and careful evaluation is warranted so an underlying androgen-secreting tumour is not overlooked [28]. The causes of ‘nontumorous hyperandrogenism’, such as PCOS and non-classic CAH (NCAH), so-called functional hyperandrogenism, are clinically manifested before menopause, and hirsutism and/or alopecia may even worsen during perimenopause and early menopause [29,30], but signs of virilization and/or uterine bleeding should always direct toward ovarian hyperandrogenism or an androgen-secreting neoplasm [28]. After menopause, androgen levels are positively associated with an increased breast cancer risk and gynaecological malignancies, but impaired bone mineral density is also seen at this age; nevertheless, there is a positive correlation between androgen levels and total bone mass [31] (Figure 1).

## 4. Hyperandrogenism in Adrenal Tumours

The prevalence of adrenocortical tumours in patients with signs or symptoms of hyperandrogenism is quite low. Several case series attest to a prevalence of 0.1–1.7% of adrenal tumours in patients screened for AE [32,33,34,35,36,37,38,39,40]. Although these data could reappraise the problem, it should be underlined that, in the setting of adrenal tumours associated with AE, malignancies represent up to 75% of cases and the second cause of AE in post-menopausal women, as assessed in a large case series [40]. On the other hand, signs and symptoms of hyperandrogenism in adrenal tumours are present in more than half of patients [41]. Androgen-secreting adrenal tumours have been associated with a rapid onset of signs and symptoms of AE, including hirsutism, virilization, anovulatory cycles, and alopecia in women, as discussed above. In males, clinical manifestations are too difficult to ascertain and it may be related to androgen–oestrogen conversion at the peripheral level, such as gynecomastia. The AE pattern in patients with an adrenal tumour seems to be characterized by a simultaneous rise in DHEAS, androstenedione, and testosterone. However, a highly predictive pattern was not identified for a diagnosis of an adrenal tumour. On the other hand, incidental-discovered adrenal mass (i.e., adrenal incidentaloma) is a common clinical finding and represents the most important adrenal disorder, and it is estimated to be present in 3–5% of the general population [41], with an age-dependent increase in prevalence [42]. Most adrenal incidentalomas are non-secreting adenomas [42], even though a slight cortisol excess could be present in 1/3 of cases. This condition is usually associated with lower androgen levels. Mild cortisol excess, also known as autonomous cortisol secretion, is characterized by low/suppressed levels of ACTH in the presence of normal–high/normal cortisol levels at 8 AM; a partial suppression of morning cortisol after an oral 1 mg overnight dexamethasone test (Nugent Test) could be present [42,43]. In this scenario, ACTH-stimulated androgen synthesis by zona reticularis could be blunted, explaining the reduction in circulating androgen levels. However, several reports provided evidence of slight androgen excess in some cases of adrenal incidentalomas. Patients with adrenal incidentaloma presented a higher response of 17-OH progesterone dynamically evaluated after an iv 250 mcg SYNACTHEN*^®^* stimulation test [44,45,46], in the absence of CAH. Surgical removal of adrenal mass seems to restore a normal response after stimulation [45]. An interpretation of this phenomenon could be related to an altered 21-hydroxylase activity of neoplastic cells as a feature of dedifferentiation. However, these data remain controversial and need to be further investigated. On the other hand, CAH is associated with increasing the incidence of adrenal incidentalomas. It has been estimated that around 0.8% of adrenal incidentalomas have been related to genetically proven *CYP21A2* gene variants with some differences according to ethnicity of the population studied [47]. Finally, although AE is a rare condition associated with adrenal tumours, it could be predictive of malignancy. A careful evaluation of the androgen pattern, also in patients with clear signs of hyperandrogenism, could be useful. A mass-spectrometry-based assay is the gold standard since it could measure all androgens simultaneously, reducing margins of inaccuracy. 

## 5. Diagnosis

The diagnosis of adrenal hyperandrogenism requires an accurate anamnestic collection, aimed at highlighting useful aspects such as the age of onset of the symptoms and the rapidity of their deterioration, and the association with other signs/symptoms of endocrine hypersecretion (such as, for example, symptoms and signs of hypercortisolism, galactorrhoea, obesity, and menstrual cycle disorders). As is well known, it will be necessary to exclude the most frequent causes of hyperandrogenism, which are PCOS and NCAH. The diagnosis of CAH is usually performed with a basal and/or dynamic evaluation of 17-OH progesterone (17OHP) in the early follicular phase: 17OHP concentration above 30 nmol/L is consistent with NCAH; however, a *CYP21A2* genetic test should be performed to confirm the diagnosis and to exclude other causes of high levels of 17OHP, such as adrenal malignancies [40]. 

Laboratory evaluation should focus on measuring total testosterone levels. According to current guidelines of the Endocrine Society, the gold standard is methods based on mass spectrometry, which allow a more accurate determination than traditional RIA or ELISA methods [48].

The evaluation of free testosterone is affected by analytical inaccuracy, and reliable values can only result from an analysis by equilibrium dialysis, practiced in a few centres [49]. Alternatively, in cases where total testosterone is within normal limits, free testosterone can be derived from calculation formulas that also require measurement of SHBG and albumin levels [50].

The finding of high levels of total testosterone (values above 5 nmol/L), however, does not clarify the origin of the hormone hypersecretion, which can derive from both the ovary and the adrenal gland. The evaluations of DHEAS and androstenedione are useful in the differential diagnosis [32].

**Figure 2:**
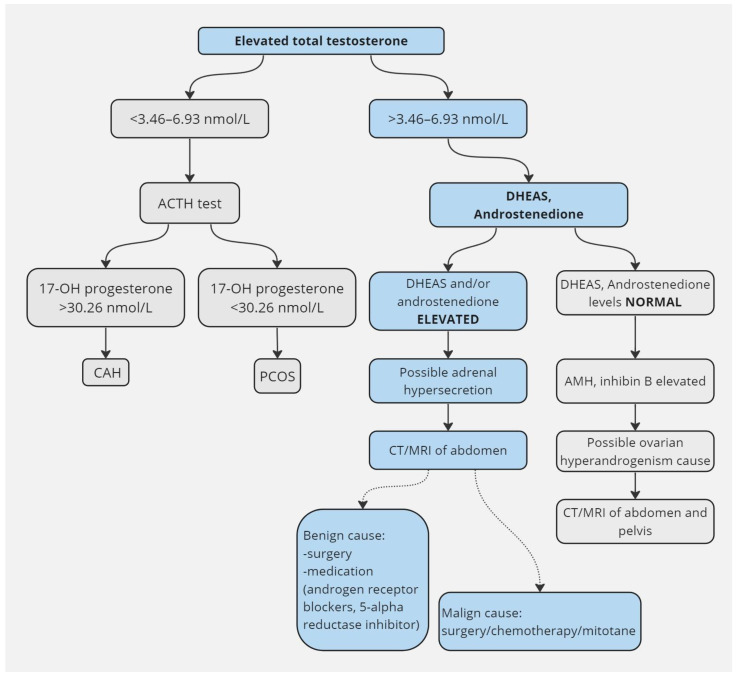
Diagnostic and therapeutic algorithm.

Typically, an androgen-producing adrenal tumour is associated with high levels of DHEA-S (more than twice the upper reference limit). The co-presence of ACTH-independent hypercortisolism in Cushing’s syndrome, which can be assessed through the evaluation of ACTH and morning cortisol, 24 h urinary cortisol, and a 1 mg dexamethasone suppression test, supports the hypothesis of adrenal hyperandrogenism. On the other hand, increased levels of the anti-müllerian hormone and inhibin B may point towards an ovarian origin [51] (Figure 2).

Discriminating the possible origin of hyperandrogenism before performing imaging investigations is of particular importance, given the relative high prevalence of adrenal incidentalomas in the general population leading to erroneous conclusions [41].

Radiological diagnostics allows you to confirm the suspected diagnosis. CT also has excellent diagnostic performance in identifying adrenal adenomas/carcinomas and represents the first choice. Magnetic resonance imaging of the abdomen and pelvis also allows the evaluation of the presence of masses in the adrenal lodges or the pelvis. The use of ultrasound is reserved for cases in which an ovarian origin of hyperandrogenism is suspected [41].

## 6. Treatment

The treatment of adrenal hyperandrogenism due to an adrenal tumour is eminently surgical, in consideration of the frequent malignant origin. According to the last guidelines of the American College of Endocrine Surgery [52], surgical management depends on the dimension of the adrenal mass, invasion of other organs (such as kidney and vena cava), or other characteristics of malignancy. In the case of larger tumours (>9 cm) and local invasion, open transabdominal adrenalectomy is performed en bloc with other organs involved by the neoplasia [41]. In the case of small tumours and no signs of local invasion, a less invasive approach (posterior retroperitoneoscopic or anterolateral laparoscopic) is preferred [41]. In the presence of adrenocortical carcinoma, surgical management should be performed in a high-volume multidisciplinary centre, in order to improve recurrence outcomes; the goal of surgical intervention is an R0 en bloc adrenal mass removal with an intact capsule [52].

After surgery, patients with high risk of recurrence, i.e., non-complete resection or patients with metastatic disease or high-grade neoplasia according to the Weiss score, should be treated with mitotane in an adjuvant/palliative setting. Mitotane targets the enzymes involved in the steroidogenic pathway, including 11β-hydroxylase and cytochrome P450 side chain cleavage, thus lowering the secretion of steroid hormones [53]. In adrenocortical carcinoma, the main effect of mitotane, besides reducing the steroid production, is tumour cell death [54]. Patients undergoing mitotane treatment develop adrenal insufficiency and should receive glucocorticoid replacement therapy. Patients with recurrence/progression under mitotane treatment, not feasible for a complete resection, or with very early progression (<6 months) are candidates for the cytotoxic regimen with Etoposide–Doxorubricin–Cisplatin, as stated by ESE/ENSAT guidelines [55].

Androgen receptor blockers, such as spironolactone (50–200 mg die) or flutamide (250 mg die), or 5-alpha reductase inhibitors such as finasteride (1–5 mg die) could be useful but in mild forms that can occasionally be associated with benign adrenal lesions [48,51,56]. All these drugs are effective in counteracting signs or symptoms of hyperandrogenism and are often combined with dermo-cosmetological solutions.

On the other hand, side effects of these medications should be considered in clinical practice. Spironolactone has a well-known dosage-dependent anti-mineralocorticoid activity: serum electrolytes and blood pressure should be carefully evaluated in treated patients; it also acts as an antagonist of the progesterone receptor and therefore it can be associated with menstrual cycle disturbance in fertile patients.

As far as 5-alpha-reductase inhibitors are concerned, hepatotoxicity is the most important side effect.

Each of these mentioned drugs have a teratogenic effect and are contraindicated in pregnant patients or those seeking pregnancy.

As far as CAH is concerned, treatment with glucocorticoids is effective to reduce signs and symptoms of hyperandrogenism and hypoadrenalism. In the case of failure with common antiandrogenic/glucocorticoid therapy, or in the presence of serious side effects, bilateral adrenalectomy has been proposed [57], although this practice is not covered by the Endocrine Society practice guidelines [58]. Recently, new glucocorticoids with modified release have been approved for treatment of CAH. They are able to better mimic the circadian rhythm of cortisol secretion in order to suppress ACTH secretion typical of this syndrome and to avoid chronic glucocorticoid excess, related to higher metabolic and cardiovascular impairments.

## 7. Conclusions

Although hyperandrogenism of an adrenal origin is an infrequent finding, it may underlie the presence of malignancy of the adrenal gland. Therefore, it must be properly investigated and diagnosed. If conditions such as CAH represent the most frequent condition of adrenal hyperandrogenism at a pre-menopausal/juvenile age, adrenal carcinoma represents an important case in post-menopause. The typical sudden onset and clinical features, coupled with suggestive laboratory and imaging findings, can lead to the correct diagnosis and treatment.

## Data Availability

Not applicable.

## References

[1] Ghizzoni L., Mastorakos G., Vottero A. (1999). Adrenal Hyperandrogenism in Children. J. Clin. Endocrinol. Metab..

[2] Yilmaz B., Yildiz B.O. (2019). Endocrinology of Hirsutism: From Androgens to Androgen Excess Disorders. Hyperandrogenism Women.

[3] Longcope C. (1986). Adrenal and gonadal androgen secretion in normal females. Clin. Endocrinol. Metab..

[4] Claahsen-van der Grinten H.L., Speiser P.W., Ahmed S.F., Arlt W., Auchus R.J., Falhammar H., Flück C.E., Guasti L., Huebner A., Kortmann B.B. (2022). Congenital Adrenal Hyperplasia-Current Insights in Pathophysiology, Diagnostics, and Management. Endocr. Rev..

[5] Orfanos C.E., Adler Y.D., Zouboulis C.C. (2000). The SAHA Syndrome. Horm. Res. Paediatr..

[6] Vigouroux C. (2010). What have we learned form monogenic forms of severe insulin resistance associated with PCOS/HAIRAN?. Ann. Endocrinol..

[7] Zouboulis C.C., Degitz K. (2004). Androgen action on human skin—from basic research to clinical significance. Exp. Dermatol..

[8] Rosol T.J., Yarrington J.T., Latendresse J., Capen C.C. (2001). Adrenal Gland: Structure, Function, and Mechanisms of Toxicity. Toxicol. Pathol..

[9] Gallo-Payet N., Battista M.-C. (2014). Steroidogenesis-Adrenal Cell Signal Transduction. Compr. Physiol..

[10] Rainey W.E., Rehman K.S., Carr B.R. (2004). The human fetal adrenal: Making adrenal androgens for placental estrogens. Semin. Reprod. Med..

[11] Marina L.V., Ivović M., Tančić-Gajić M., Arizanović Z., Raković D., Milin-Lazović J., Kendereški A., Micić D., Vujović S. (2018). Luteinizing hormone and insulin resistance in menopausal patients with adrenal incidentalomas: The cause-effect relationship?. Clin. Endocrinol..

[12] Bienenfeld A., Azarchi S., Lo Sicco K., Marchbein S., Shapiro J., Nagler A.R. (2018). Androgens in Women: Androgen mediated skin disease and patient evaluation (Part I). J. Am. Acad. Dermatol..

[13] Witchel S.F. (2017). Congenital Adrenal Hyperplasia. J. Pediatr. Adolesc. Gynecol..

[14] Turcu A.F., Auchus R.J. (2015). Adrenal Steroidogenesis and Congenital Adrenal Hyperplasia. Endocrinol. Metab. Clin..

[15] Nguyen A.D., Conley A.J. (2008). Adrenal Androgens in Humans and Nonhuman Primates: Production, Zonation and Regulation. Endocr. Dev..

[16] KCollomp C., Buisson F., Lasne R. (2015). Collomp. DHEA, physical exercise and doping. J. Steroid Biochem. Mol. Biol..

[17] Dharia S., Parker C.R. (2014). Adrenal Androgens and Aging. Semin. Reprod. Med..

[18] Lizneva D., Gavrilova-Jordan L., Walker W., Azziz R. (2016). Androgen excess: Investigations and management. Best Pract. Res. Clin. Obstet. Gynaecol..

[19] Charmandari E., Kino T. (2010). Chrousos syndrome: A seminal report, a phylogenetic enigma and the clinical implications of glucocorticoid signalling changes. Eur. J. Clin. Investig..

[20] Pugeat M., Raverot G., Plotton I., Brac de la Perrière A., Mirakian P., Déchaud H., Berger N., Peix J.L., Contemporary Endocrinology. Azziz R., Nestler J.E., Dewailly D. (2006). Androgen-Secreting Adrenal and Ovarian Neoplasms. Androgen Excess Disorders in Women.

[21] da Cunha M.G., Fonseca F.L., Machado C.D. (2013). Androgenic hormone profile of adult women with acne. Dermatology.

[22] Wang Y.Y., Li S.W., Luo S., Qin L., Zeng X., Li L., Li X.H. (2019). How to evaluate acne in reproductive- age women: An epidemiological study in Chinese communities. BioMed Res. Int..

[23] Cussen L., McDonnell T., Bennett G., Thompson C.J., Sherlock M., O’Reilly M.W. (2022). Approach to androgen excess in women: Clinical and biochemical insights. Clin. Endocrinol..

[24] Carmina E., Dreno B., Lucky W.A., Agak W.G., Dokras A., Kim J.J., Lobo R.A., Ramezani Tehrani F., Dumesic D. (2022). Female Adult Acne and Androgen Excess: A Report From the Multidisciplinary Androgen Excess and PCOS Committee. J. Endocr. Soc..

[25] Kumarendran B., O’Reilly M.W., Manolopoulos K.N., Toulis K.A., Gokhale K.M., Sitch A.J., Wijeyaratne C.N., Coomarasamy A., Arlt W., Nirantharakumar K. (2018). Polycysticovary syndrome, androgen excess, and the risk of nonalcoholic fattyliver disease in women: A longitudinal study based on a UnitedKingdom primary care database. PLoS Med..

[26] Kumarendran B., Sumilo D., O’Reilly M.W., Toulis K.A., Gokhale K.M., Wijeyaratne C.N., Coomarasamy A., Arlt W., Tahrani A.A., Nirantharakumar K. (2019). Increased risk ofobstructive sleep apnoea in women with polycystic ovary syndrome: Apopulation-based cohort study. Eur. J. Endocrinol..

[27] Moran L.J., Misso M.L., Wild R.A., Norman R.J. (2010). Impaired glucosetolerance, type 2 diabetes and metabolic syndrome in polycysticovary syndrome: A systematic review and meta-analysis. Hum. Reprod. Update.

[28] Markopoulos M.C., Kassi E., Alexandraki K.I., Mastorakos G., Kaltsas G. (2015). Hyperandrogenism after menopause. Eur. J. Endocrinol..

[29] Rothman M.S., Wierman M.E. (2011). How should postmenopausal androgen excess be evaluated?. Clin. Endocrinol..

[30] Markopoulos M.C., Rizos D., Valsamakis G., Deligeoroglou E., Grigoriou O., Chrousos G.P., Creatsas G., Mastorakos G. (2011). Hyperandrogenism in women with polycystic ovary syndrome persists after menopause. J. Clin. Endocrinol. Metab..

[31] Vanderschueren D., Vandenput L., Boonen S., Lindberg M.K., Bouillon R., Ohlsson C. (2004). Androgens and bone. Endocr. Rev..

[32] Waggoner W., Boots L.R., Azziz R. (1999). Total testosterone and DHEAS levels as predictors of androgen-secreting neoplasms: A populational study. Gynecol. Endocrinol..

[33] Azziz R., Sanchez L.A., Knochenhauer E.S., Moran C., Lazenby J., Stephens K.C., Taylor K., Boots L.R. (2004). Androgen excess in women: Experience with over 1000 consecutive patients. J. Clin. Endocrinol. Metab..

[34] Glintborg D., Henriksen J.E., Andersen M., Hagen C., Hangaard J., Rasmussen P.E., Schousboe K., Hermann A.P. (2004). Prevalence of endocrine diseases and abnormal glucose tolerance tests in 340 Caucasian premenopausal women with hirsutism as the referral diagnosis. Fertil. Steril..

[35] Unluhizarci K., Gokce C., Atmaca H., Bayram F., Kelestimur F. (2004). A detailed investigation of hirsutism in a Turkish population: Idiopathic hyperandrogenemia as a perplexing issue. Exp. Clin. Endocrinol. Diabetes.

[36] Carmina E., Rosato F., Jannì A., Rizzo M., Longo R.A. (2006). Extensive clinical experience: Relative prevalence of different androgen excess disorders in 950 women referred because of clinical hyperandrogenism. J. Clin. Endocrinol. Metab..

[37] Escobar-Morreale H.F., Sanchón R., San Millán J.L. (2008). A prospective study of the prevalence of nonclassical congenital adrenal hyperplasia among women presenting with hyperandrogenic symptoms and signs. J. Clin. Endocrinol. Metab..

[38] Fanta M., Cibula D., Vrbíková J. (2008). Prevalence of nonclassic adrenal hyperplasia (NCAH) in hyperandrogenic women. Gynecol. Endocrinol..

[39] Karrer-Voegeli S., Rey F., Reymond M.J., Meuwly J.Y., Gaillard R.C., Gomez F. (2009). Androgen dependence of hirsutism, acne, and alopecia in women: Retrospective analysis of 228 patients investigated for hyperandrogenism. Medicine.

[40] Elhassan Y.S., Idkowiak J., Smith K., Asia M., Gleeson H., Webster R., Arlt W., O’Reilly M.W. (2018). Causes, Patterns, and Severity of Androgen Excess in 1205 Consecutively Recruited Women. J. Clin. Endocrinol. Metab..

[41] Fassnacht M., Arlt W., Bancos I., Dralle H., Newell-Price J., Sahdev A., Tabarin A., Terzolo M., Tsagarakis S., Dekkers O.M. (2016). Management of adrenal incidentalomas: European Society of Endocrinology Clinical Practice Guideline in collaboration with the European Network for the Study of Adrenal Tumors. Eur. J. Endocrinol..

[42] Sherlock M., Scarsbrook A., Abbas A., Fraser S., Limumpornpetch P., Dineen R., Stewart P.M. (2020). Adrenal Incidentaloma. Endocr. Rev..

[43] Ueland G., Grinde T., Methlie P., Kelp O., Løvås K., Husebye E.S. (2020). Diagnostic testing of autonomous cortisol secretion in adrenal incidentalomas. Endocr. Connect..

[44] Seppel T., Schlaghecke R. (1994). Augmented 17 alpha-hydroxyprogesterone response to ACTH stimulation as evidence of decreased 21-hydroxylase activity in patients with incidentally discovered adrenal tumours (‘incidentalomas’). Clin. Endocrinol..

[45] Tóth M., Racz K., Adleff V., Varga I., Fütö L., Jakab C., Karlinger K., Kiss R., Gláz E. (2000). Comparative analysis of plasma 17-hydroxyprogesterone and cortisol responses to ACTH in patients with various adrenal tumors before and after unilateral adrenalectomy. J. Endocrinol. Investig..

[46] Terzolo M., Osella G., Ali A., Borretta G., Magro G.P., Termine A., Paccotti P., Angeli A. (1996). Different patterns of steroid secretion in patients with adrenal incidentaloma. J. Clin. Endocrinol. Metab..

[47] Falhammar H., Torpy D.J. (2016). Congenital adrenal hyperplasia due to 21-hydroxylase deficiency presenting as adrenal incidentaloma: A systematic review and meta-analysis. Endocr. Pract..

[48] Martin K.A., Chang R.J., Ehrmann D.A., Ibanez L., Lobo R.A., Rosenfield R.L., Shapiro J., Montori V.M., Swiglo B.A. (2018). Evaluation and Treatment of Hirsutism in Premenopausal Women: An Endocrine Society Clinical Practice Guideline. J. Clin. Endocrinol. Metab..

[49] Keevil B.G., Adaway J. (2019). Assessment of free testosterone concentration. J. Steroid Biochem. Mol. Biol..

[50] Vermeulen A., Verdonck L., Kaufman J.M. (1999). A critical evaluation of simple methods for the estimation of free testosterone in serum. J. Clin. Endocrinol. Metab..

[51] Yoldemir T. (2022). Postmenopausal hyperandrogenism. Climacteric.

[52] Yip L., Duh Q.Y., Wachtel H., Jimenez C., Sturgeon C., Lee C., Velázquez-Fernández D., Berber E., Hammer G.D., Bancos I. (2022). American Association of Endocrine Surgeons Guidelines for Adrenalectomy: Executive Summary. JAMA Surg..

[53] (2013). Lehmann TP, Wrzesiński T and Jagodziński PP: The effect of mitotane on viability, steroidogenesis and gene expression in NCI-H295R adrenocortical cells. Mol. Med. Rep..

[54] Corso C.R., Acco A., Bach C., Bonatto S.J.R., de Figueiredo B.C., de Souza L.M. (2021). Pharmacological profile and effects of mitotane in adrenocortical carcinoma. Br. J. Clin. Pharmacol..

[55] Fassnacht M., Dekkers O.M., Else T., Baudin E., Berruti A., de Krijger R., Haak H.R., Mihai R., Assie G., Terzolo M. (2018). European Society of Endocrinology Clinical Practice Guidelines on the management of adrenocortical carcinoma in adults, in collaboration with the European Network for the Study of Adrenal Tumors. Eur. J. Endocrinol..

[56] Mancini A., Bruno C., Vergani E., D’abate C., Giacchi E., Silvestrini A. (2021). Oxidative stress and low-grade inflammation in polycystic ovary syndrome: Controversies and new insights. Int. J. Mol. Sci..

[57] MacKay D., Nordenström A., Falhammar H. (2018). Bilateral Adrenalectomy in Congenital Adrenal Hyperplasia: A Systematic Review and Meta-Analysis. J. Clin. Endocrinol. Metab..

[58] Speiser P.W., Azziz R., Baskin L.S., Ghizzoni L., Hensle T.W., Merke D.P., Meyer-Bahlburg H.F., Miller W.L., Montori V.M., Oberfield S.E. (2018). Congenital Adrenal Hyperplasia Due to Steroid 21-Hydroxylase Deficiency: An Endocrine Society Clinical Practice Guideline. J. Clin. Endocrinol. Metab..

